# Modelling of packed bed and coated wall microreactors for methanol steam reforming for hydrogen production

**DOI:** 10.1039/d0ra06834a

**Published:** 2020-11-13

**Authors:** Sanaa Hafeez, Elsa Aristodemou, George Manos, S. M. Al-Salem, Achilleas Constantinou

**Affiliations:** Division of Chemical & Energy Engineering, School of Engineering, London South Bank University London SE1 0AA UK constaa8@lsbu.ac.uk +44 (0)20 7815 7185; Department of Chemical Engineering, University College London London WCIE 7JE UK; Environment & Life Sciences Research Centre, Kuwait Institute for Scientific Research P. O. Box: 24885 Safat 13109 Kuwait; Department of Chemical Engineering, Cyprus University of Technology 57 Corner of Athinon and Anexartisias 3036 Limassol Cyprus

## Abstract

A Computational Fluid Dynamics (CFD) study has been conducted to assess the performance of packed bed and coated wall microreactors for the steam reforming of methanol with a CuO/ZnO/Al_2_O_3_ based catalyst (BASF F3-01). The results obtained were compared to experimental data from the literature to assess the validity and robustness of the models, and a good validation has been obtained. The performance of the packed bed and coated wall microreactors is similar at a constant reforming temperature. It was found that methanol conversion is enhanced with increasing temperature, residence time, steam to methanol ratio, and catalyst coating thickness. Furthermore, internal and external mass transfer phenomena were investigated using the models, and it was found that there were no internal and external mass transfer resistances for this reactor configuration. Further studies demonstrated that larger catalyst pellet sizes led to the presence of internal mass transfer resistance, which in turn causes lower methanol conversions. The CFD models have exhibited a sound agreement with the experimental data, hence they can be used to predict the steam reforming of methanol in microreactors.

## Abstract 2

A Computational Fluid Dynamics (CFD) study has been conducted to assess the performance of packed bed and coated wall microreactors for the steam reforming of methanol with a CuO/ZnO/Al_2_O_3_ based catalyst (BASF F3-01).

## Graphical abstract

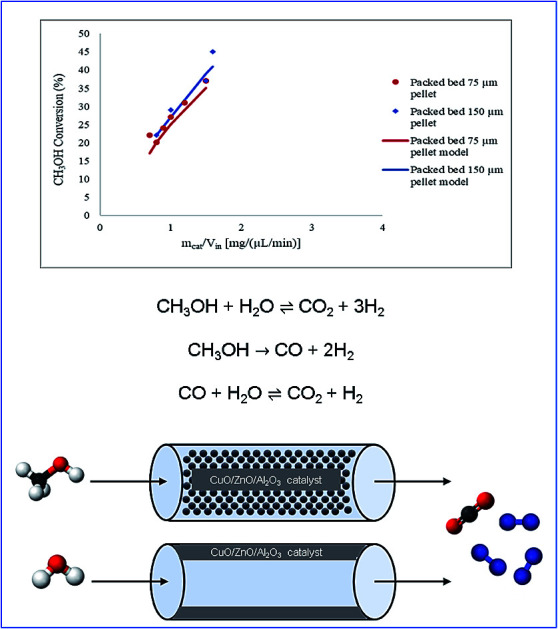

## 1. Introduction

The continuous increase of the global population has led to a decrease in the availability of conventional fossil fuels. As a result, it has become imperative to find clean renewable sources of energy to meet the current demands. Hydrogen (H_2_) is perceived to be one of the most promising alternative fuels because it is inexhaustible and is an efficient source of clean energy.^1^ However, one of the main problems faced with the widespread use of H_2_ in portable applications is its unsafe transport and distribution. A solution to this is the processing of liquid fuels to be converted into H_2_. Methanol (CH_3_OH) is an attractive choice of fuel due to its abundancy as a fuel and the fact that it can be produced from renewable resources. Further benefits of using methanol include, mild operating conditions required for its catalytic transformation into H_2_ (steam reforming), high hydrogen to carbon ratio, economical and safe handling, and its well-established production market.^2^

Methanol can be manufactured from carbon concentrated resources such as natural gas, coal or biomass; and from carbon dioxide (CO_2_) from flue gases of power plants fuelled by fossil fuels or cement factories and the atmosphere.^3^ The production of methanol from natural gas is the most popular with greater than 75% of the methanol currently being produced in this way.^4^ Here, the methanol is produced from natural gas through a syngas production route. The steam reforming of methane produces syngas (a mixture of CO_2_, CO and H_2_), and the syngas is then further upgraded to methanol typically at 200–300 °C over Cu/ZnO/Al_2_O_3_ catalysts.^5^ The production of methanol from coal process is likeable to the natural gas reforming route, whereby syngas is first produced by the gasification of coal and then the synthesis of the methanol. However, the syngas produced *via* this route has a lower H_2_ content.^3^

Proton exchange membrane fuel cells (PEMFCs) are thought to be an effective solution to current issues faced with using conventional fuels as energy sources. They present numerous benefits of no pollution, high energy density and higher energy conversion efficiency. However, the direct storage and use of hydrogen on PEMFC vehicles has a few constraints, for example, the high cost of hydrogen storage and stringent safety requirements limit the large-scale application of PEMFCs. Therefore, on-line hydrogen production using the microreactor technology effectively addresses the drawbacks of PEMFCs because it can reduce costs and comply with safety requirements.

Microreactors have been employed for renewable fuel production due to certain benefits, such as enhanced mass transfer, better temperature control leading to improved heat transfer and larger surface-area-to-volume ratios.^6,7^ Recently microreactors for hydrogen production, from hydrocarbons, have been employed to provide the on-line hydrogen source for polymer electrolyte membrane fuel cells (PEMFCs). The advantages of microreactors make them desirable for highly exothermic and fast reactions.^7–10^ As a result, microreactors have demonstrated a promising outlook for hydrogen production.^11^ The type of microreactor used for methanol steam reforming reaction significantly influences the fuel conversion and reaction efficiency. Some of the microreactors often used for methanol steam reforming are, laminated plate structure, packed bed, coated wall, silicon-chip based structure, suspended membrane structure, honeycomb structure and plate fin structure.

Packed bed microreactors for methanol reforming allow the use of commercial catalysts with moderate cost, improved catalyst availability and reproducibility, and a greater understanding of catalyst performance which is valuable in industry.^12^ Zhuang *et al.*^13^ developed a novel multichannel packed bed microreactor with bifurcation inlet manifold and rectangular outlet manifold for the steam reforming of methanol. The results show that the increase of the steam-to-methanol ratio and temperature, as well as decrease of the weight hourly space velocity and catalyst particle size, both improve the methanol conversion. The CO concentration decreases as the steam-to-methanol ratio and weight hourly space velocity increase as well as the temperature and catalyst particle size decrease.

Karim *et al.*^14^ investigated the methanol steam reforming reaction in a packed bed reactor using the commercial CuO/ZnO/Al_2_O_3_ catalyst. The focus of the study was to assess the impact of deviations from isothermal behaviours in packed bed reactors on the rates of methanol steam reforming. Initial experiments with catalyst dilution suggested higher apparent rate constants as the catalyst was diluted, indicating heat transfer limitations in the bed. The reactor diameter was therefore varied from 4.1 to 1 mm to enhance the heat transfer. The smaller diameter reactor showed higher apparent catalyst activity. The heat transfer limitations result in a temperature gradient of up to 40 K in the 4.1 mm reactor, as opposed to the 1 mm reactor which suffered from temperature variations of up to 22 K. Given that packed bed reactors are mainly used to produce H_2_ by methanol steam reforming, it is crucial to recognise the role of these heat transfer limitations. Transport limitations can result in falsified kinetics and lowered catalyst productivity.

A further study conducted by Karim *et al.*^15^ demonstrated the comparison between packed bed and coated wall microreactors. Different dimensions of both reactor configurations were tested, and the transport limitations were investigated using 2D reactor models. The dimensions of the packed bed reformer varied from 4.1 mm to 1 mm, and the results showed that temperature gradients of up to 40 K were present in the bed. Nonetheless, the coated wall microreactor was found to be devoid of any mass or heat transfer limitations in dimensions from 4.1 mm down to 200 μm. The modelling results showed that the reactor volumetric productivity increases with thicker catalyst wall coatings for a constant reactor diameter. To conclude the coated wall microreactor offers a better result to attain low pressure drops and enhanced catalyst activity compared to a packed bed microreactor.

Chougule and Sonde^16^ developed a comprehensive mathematical model to study the steam reforming of methanol in a catalytic packed-bed tubular reactor using a CuO/ZnO/Al_2_O_3_ catalyst. The model was simulated using Engineering Equation Solver (EES). Mass and heat transfer were analysed along the reformer length, to study the chemical kinetics of the reforming process. The effect of different design and operating parameters on methanol conversion and CO concentration was further investigated. The results showed that 16 parallel tubular reactor arrays of same configuration should be used for the design of methanol reformer for 5 kWe HT-PEMFC application. Designing a combined HT-PEMFC and methanol reformer system requires special attention due to the elevated operating temperatures, as the reformer behaves differently under different conditions, understanding the effect of these parameters is essential for making optimal design compromises, proper heat integration and control strategies to achieve a reliable and efficient fuel cell system.

To further understand the methanol steam reforming reaction for hydrogen production, numerical modelling studies have been performed in recent years. Chiu *et al.*^17^ adopted CFD software to analyse the performance of the methanol steam reforming process in a tubular packed-bed reactor. The model consisted of chemical and physical parameters, as well as operating variables, and was used to investigate the individual influences on the hydrogen production efficiency. Moreover, the dimensionless Damköhler number was suggested to be an important index that quantitatively measured the performance of an MSR process.

Zhuang *et al.*^18^ numerically investigated a multichannel reactor with a bifurcation inlet manifold, a rectangular outlet manifold, and sixteen parallel minichannels with commercial CuO/ZnO/Al_2_O_3_ catalyst for methanol steam reforming. The effects of steam to carbon molar ratio, weight hourly space velocity, operating temperature and catalyst layer thickness on the methanol steam reforming performance were evaluated and discussed. The results showed that an operating temperature of 548 K, steam to carbon ratio of 1.3, and weight hourly space velocity of 0.67 h^−1^ are recommended operating conditions for methanol steam reforming by reactor with catalyst fully packed in the parallel minichannels.

Ghasemzadeh *et al.*^2^ performed a theoretical study to evaluate the performance of silica and Pd–Ag membrane reactors at the same operating conditions and reaction kinetics for hydrogen production from methanol steam reforming. A CFD model was developed, firstly validating a traditional reactor with experimental literature data. The effects of reaction pressure and temperature on the reactor's performance in terms of hydrogen yield, methanol conversion and CO selectivity were hence studied and discussed. The results showed that the silica membrane reactor results showed the best performance over the Pd–Ag MR and the TR as well, demonstrating optimum results at 513 K, 10 bar, sweep-factor = 6, GHSV = 6000 h^−1^ and feed molar ratio = 3/1 with CO selectivity equal to 0.04%, methanol conversion and hydrogen yield >90%.

Heidarzadeh and Taghizadeh^19^ performed a CFD study for hydrogen production in an annular microchannel reactor coated with CuO/ZnO/Al_2_O_3_ catalyst. The modelling mechanism included methanol reforming reaction, methanol decomposition, and water-gas shift reaction. Furthermore, the effects of temperature variations were examined, and the conducted surveys were compared with the experimental results. The simulation results were in good agreement with the experimental data and showed that temperature increases at various feed flow rates would lead to enhanced amounts of CO and CO_2_, while at a constant temperature, the amounts of hydrogen and CO and CO_2_ decrease with increasing feed flow rates.

Performing numerical studies using CFD software is valuable as it provides an understanding of parameter optimisation for the steam reforming of methanol for hydrogen production. The modelling of microreactor systems for hydrogen/fuel production is not well established, contrary to larger scale systems, adding to the novelty of this work. In the current study, the steam reforming of methanol over a CuO/ZnO/Al_2_O_3_ based catalyst (BASF F3-01) is investigated and presented in this study using 2-D packed bed and coated wall microreactors. Computational Fluid Dynamic (CFD) methodologies were used to model the transport phenomena and the thermal properties of the gas mixture associated with the composition of each species throughout the reformer. Parameters such as, the size of catalyst particle and the wall coating thickness were investigated to assess their effects of product composition and methanol conversion, and further studies based on internal and external transport limitations are additionally performed. A validation of the microreactor models with the experimental data is exhibited and a very good agreement was observed between the CFD microreactor models and the experimental data from literature.^20^

The comprehensive CFD models created in this work are a valuable tool for understanding which parameters can potentially optimise the methanol steam reforming process and can successfully predict the steam reforming of methanol in microreactors. The heterogeneous 2-phase catalytic models give rise to the study of particle fluid transport phenomena which provides an understanding of internal and external mass transfer limitations, as opposed to the common pseudo homogeneous models. The models can be compared to experimental data from literature to understand which parameters lead to internal diffusion limited reactions, which can often be time consuming and expensive when performed on an experimental basis.

## 2. Modelling methodology

CFD was used to simulate the isothermal microreactors (packed bed and coated wall) and to determine the particle-fluid transport phenomena occurring in the microreactors. Experimental studies can often be laborious and costly, whereas CFD studies can effortlessly provide elaborate details with minimal effort on the space-time variations regarding reactant flows, concentrations, and temperatures within the reactor. As a result, CFD is deemed a favourable methodology to use when estimating parameters and enables a comprehensive study of the physiochemical processes used.^21^ The software used to solve the study has CFD as an integrated methodology in the modules used. The 2-D modelling methodology was adopted as it enhances the accuracy of the microreactor modelling and demonstrates a truer reflection of the actual reactor geometry.

### 2.1 Reaction kinetics & pathway

The steam reforming of methanol reaction has been studied extensively, and several kinetic models have been suggested. The model described by Amphlett *et al.*^22^ was used for the experimental work^20^ and describes the steam reforming of methanol using the same CuO/ZnO/Al_2_O_3_ catalyst in a packed bed reactor. This model will be used throughout the study. The catalytic steam reforming of methanol occurs by an overall reaction with the CuO/ZnO/Al_2_O_3_ catalyst:^22^1
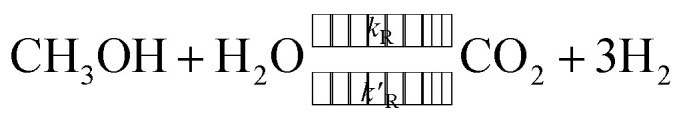


A proportion of the methanol also decomposes to CO by:2
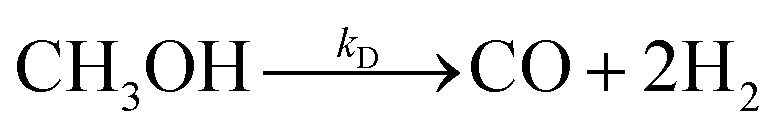


Under certain conditions, the water–gas-shift reaction can have a notable effect on the composition of the product gas:3
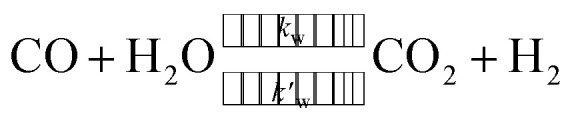
where the subscripts R, D and W denote the reforming, decomposition, and water–gas-shift reactions, respectively. For a range of conditions utilised to produce hydrogen for transportation purposes, reactions (1) and (2) can be considered irreversible because the equilibrium conversion of methanol is often 100%. In addition, the water–gas-shift reaction can be neglected without a significant loss in accuracy.^22^ The reaction rate expressions for the reforming (*r*_R_) and decomposition (*r*_D_) reactions can be found as follows:

4 *r*_R_ = *k*_R_*c*_M_

5
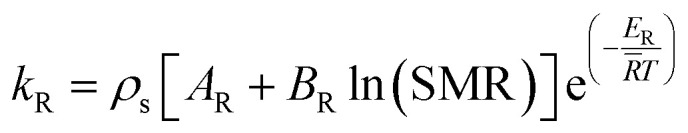

6 *r*_D_ = *k*_D_

7
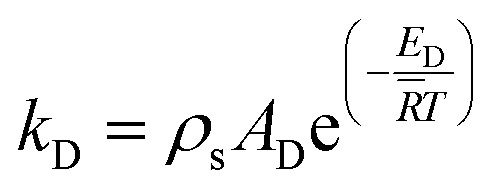
where *k*_R_ and *k*_D_ are the reaction rate constants for the reforming and decomposition reactions respectively, and *ρ*_s_ is the density of the solid catalyst. *A*_R_, *B*_R_ and *A*_D_ are Amphletts constants,^22^ SMR is the molar ratio of steam to methanol, and *E*_R_ and *E*_D_ are the activation energy for the reforming and decomposition reactions, respectively. Based on reactions (4) and (6) above, the following set of expressions can be obtained for the generation rates of the species:8
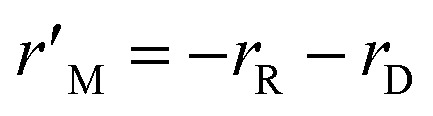
9
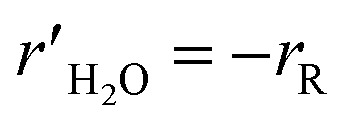
10
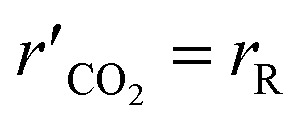
11
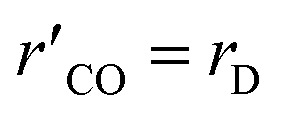
12
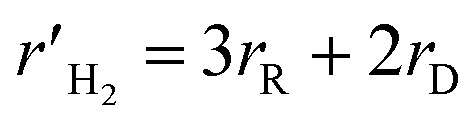


### 2.2 Numerical procedure and conservation equations

**Fig. 1:**
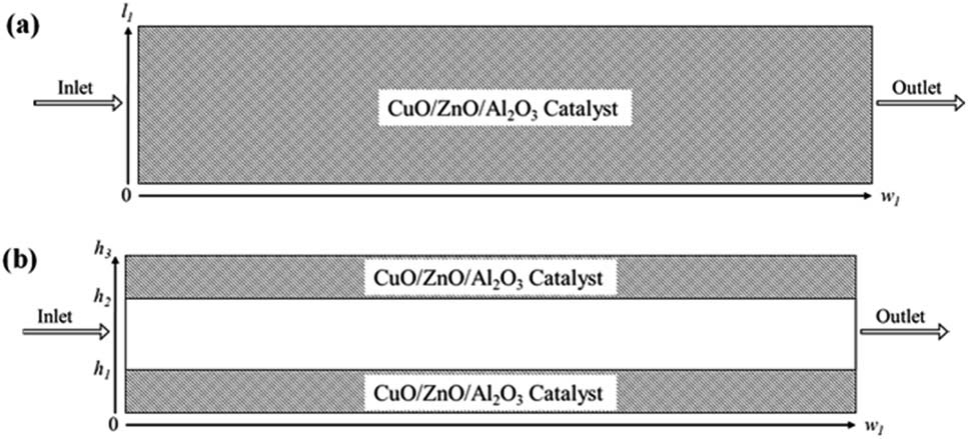
Schematic representations of the (a) packed bed microreactor; and (b) coated wall microreactor used for the CFD study.

2-D microreactor models were simulated based on the following assumptions: (a) the concentration and temperature gradients only occur in the axial and radial directions; (b) the methanol-steam mixture flow is presumed to be steady state and radially uniform throughout the packed catalyst bed; (c) the ideal gas law is applicable for the gas species in the microreactor; (d) the axial fluid velocity is constant with uniform physical properties and transport coefficients; (e) laminar flow conditions were also investigated; (f) 3-D methods are also employed; and (g) non-isothermal behaviour is also studied in the microreactors. The catalyst used in both the packed bed and coated wall reactors is a CuO/ZnO/Al_2_O_3_ based catalyst (BASF F3-01), and it is understood that the gas species react heterogeneously with the catalyst. Two catalyst sizes are investigated for the packed bed reformer, 75 and 150 μm, with loading lengths of 1.1 and 0.9 cm, respectively. The height of the microreactor is 1.5 mm.^20^ For the coated wall microreactor, the length of the catalyst coating layer is 2–6 cm, with an average coating thickness of 100 μm.^20^Fig. 1 displays schematic diagrams of the rectangular 2-D packed bed and coated wall microreactors used in this study.

The methanol reforming reaction (reaction (1)) occurs on the solid surface of the catalyst particle in the packed bed reactor. The heterogeneous reaction rate is inclusive of the mass and heat transfer, which occurs in the porous medium of the catalyst bed. The heterogeneous reaction rate is given by:^23,24^13
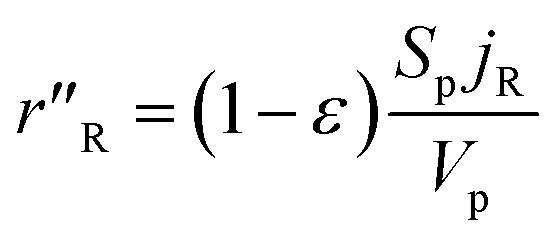
where *S*_p_ is the specific surface area of the catalyst pellet in the catalyst packed bed, *V*_p_ is the volume of the solid spherical pellet, and *j*_R_ is the molar flux of the methanol at the surface of the solid pellet. The reacting fluids encounter a convective resistance between the bulk fluid and the solid surface, and a diffusive resistance which occurs within the solid particle. The molar flux is given by:14
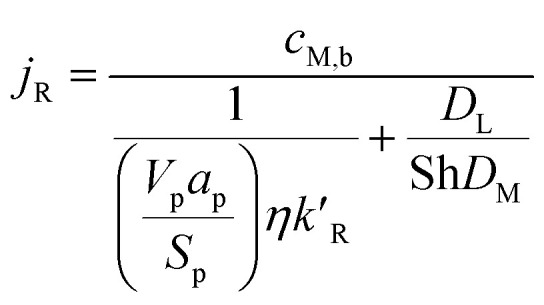
where *c*_M,b_ is the bulk concentration of methanol in the free stream between the solid particles, *a*_p_ is the ratio of the porous surface area of the pellet per unit volume of the pellet, *D*_M_ is the molecular diffusion coefficient, and *η* is the particle effectiveness factor. The characteristic diffusion length (*D*_L_) is given by:15
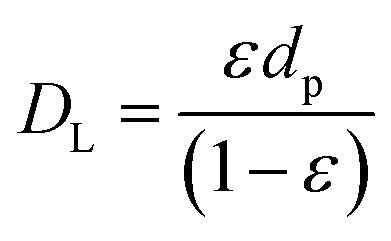
where *d*_p_ is the diameter of the catalyst pellet. The kinetic rate constant 
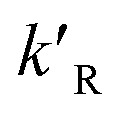
 for the reaction is related to the rate constant in eqn (4) and can be found as:16
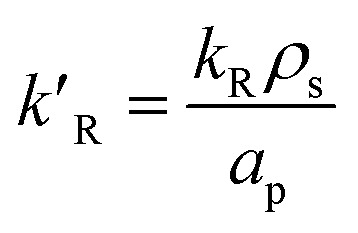


The effectiveness factor, *η*, is defined as the ratio of the observed rate to the rate that would be found if there were no internal diffusion limitations. For a first-order reaction in a spherical catalyst particle, an expression for the effectiveness factor can be derived as:^25^17
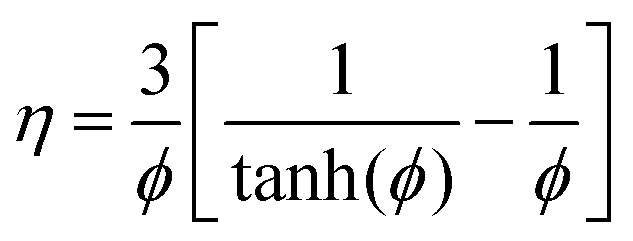
where *ϕ* is the Thiele modulus and is given by:^26^18
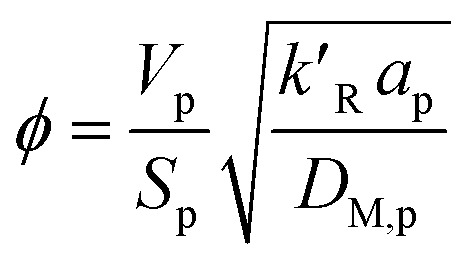


The effective diffusivity within the catalyst particle *D*_M,p_ is based on the ordinary bulk diffusivity *D*_M_ and the Knudsen diffusivity *D*_K_, calculated by:^27^19
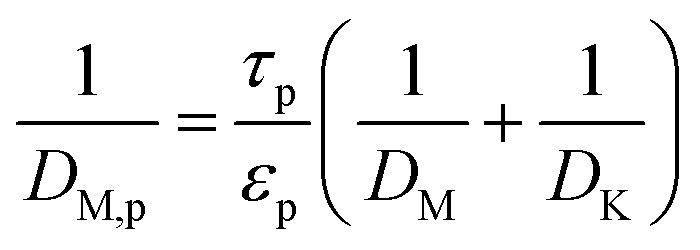


The ordinary bulk diffusivity *D*_M_ can be calculated using the Wilke model for multicomponent mass diffusion of dilute gases:^28^20
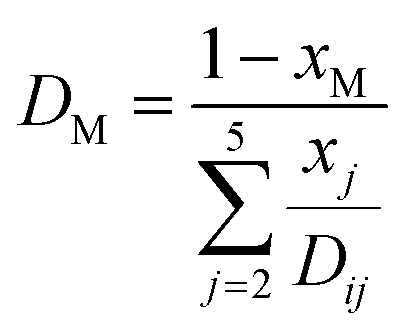


Further, *D*_M_ is the effective multicomponent diffusion coefficient and assumes diffusion of the component into a multicomponent mixture of stagnant gases. The symbol *D*_*ij*_ denotes the Maxwell–Stefan diffusivities:^29^21
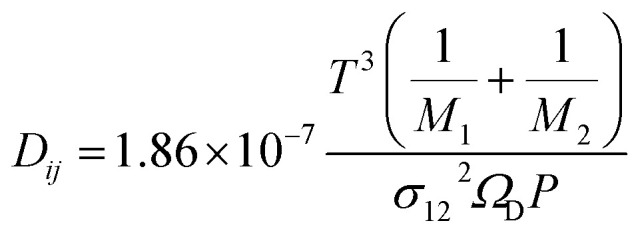
where *σ*_12_ and *Ω*_D_ are Lennard-Jones parameters and *P* is pressure in atmospheres. The Knudsen diffusivity is given by:22
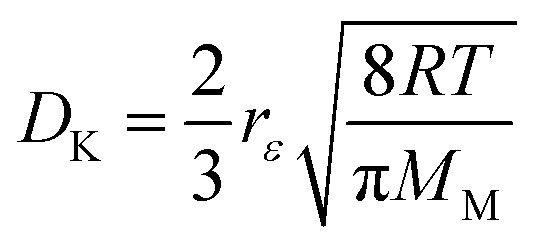


The mass balance equation for the species in the catalyst bed is given by:23
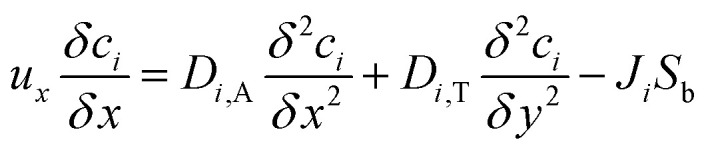
where, *u* is the fluid velocity in the axial direction, *D*_*i*,A_ and *D*_*i*,T_ are the axial and transverse diffusion coefficients respectively, *J*_*i*_ is the molar flux of *i* into the catalyst particles in mol m^−2^ s^−1^, *S* is the specific surface area of the pellets exposed to the fluids in the packed bed and can be expressed as:^30^

24 *S* = *S*_a_(1 − *ε*)

where, *ε* is the fractional voidage of the packed bed and *S*_a_ is the specific surface area, in *m*, of the particles. For spherical particles this is given by:25
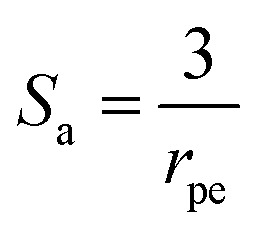
where, *r*_pe_ is the catalyst particle radius.

Along the fluid-particle boundary into the particle there is a mass flux which can be rate determined by accounting for the resistance to the mass transfer on the bulk reactants side. This can be demonstrated as:

26 *J*_*i*_ = *h*_*i*_(*c*_*i*_ − *c*_*i*,ps_)

27
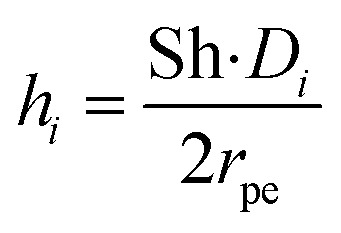
28
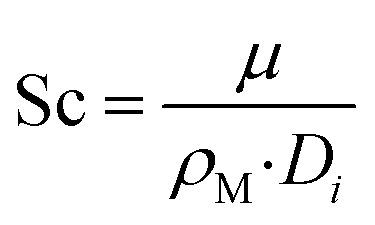
29
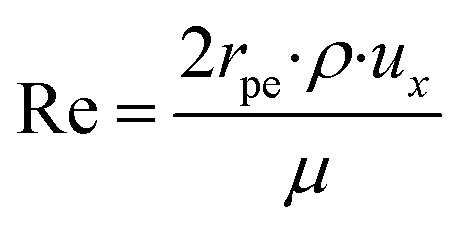

30 Sh = 2 + 0.552Re^1/2^Sc^1/3^

where, *c*_*i*,p_ is the concentration of reactant *i* at the surface of the catalyst pellet and *h*_*i*_ is the external mass transfer coefficient. The Schmidt number Sc, Reynolds number Re and the Sherwood number Sh (founded on the Frössling^31^ correlation) are dimensionless parameters which represent the mass transfer in a spherical particle,^32^ which is applicable in this work. *μ* and *ρ* represent the viscosity and density of the fluids, respectively. The density of the gas mixture *ρ*_g_ is defined as the mass-weighted average of the densities of the species if the mixture fulfils the ideal gas law and can be conveyed as follows:31
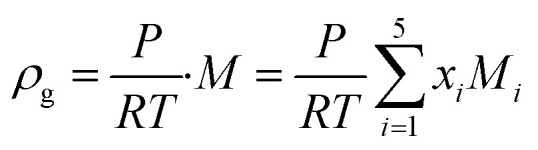


The viscosity *μ* of the gas mixture can be expressed using Wilkes mixture rule^33^ as follows:32
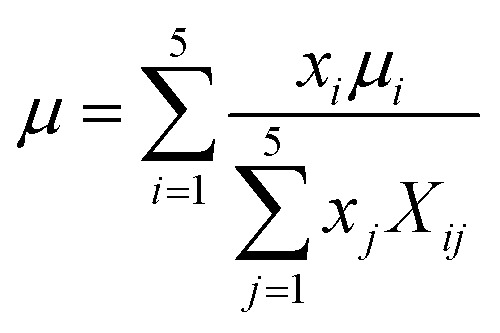
33
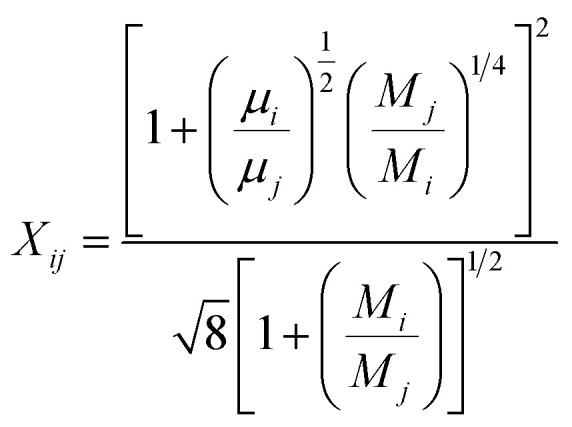


The chemical reaction takes place within the pellets and is integrated into the mass balances with the reactive pellet bed component in COMSOL®. This component has a predefined 1-D additional dimension on the normalised radius of the catalyst particle (*r* = *r*_dim_/*r*_pe_). The mass balance inside the catalyst pellet is acquired by conducting a shell balance across a spherical shell:34
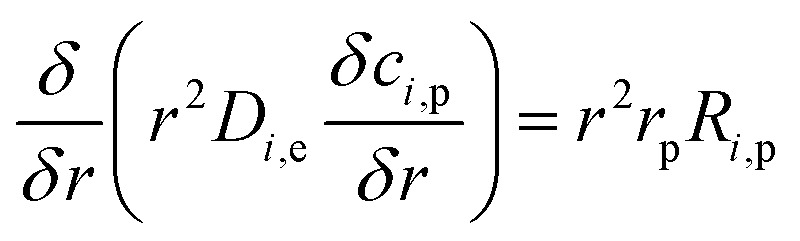
where *r* is the catalyst particle radius, *D*_*i*,e_ represents the effective diffusion coefficient of the reactant *i* within the pores of the pellet, *c*_*i*,p_ is the concentration of reactant *i* in the pellet in mol m^−3^. *R*_*i*,p_ is the reaction term.

The Navier–Stokes equations were used to model the hydrodynamics of the microreactors:35
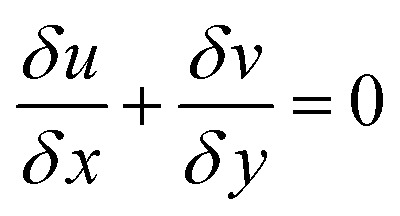
36
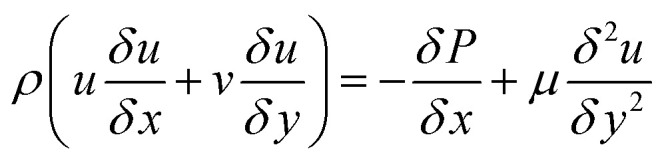
37
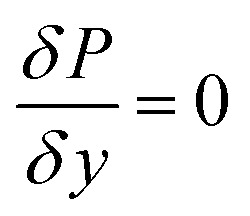


To test the assumption of isothermal behaviour in the microreactors, non-isothermal conditions were utilised to compare the findings. The energy balance for the microreactors is based on the thermal equilibrium between the two gas and solid phases, and can be expressed as:38
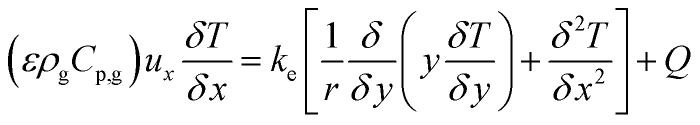
where *Q* is the energy source term and *k*_e_ is the effective thermal conductivity of the catalyst bed and is obtained using

39 *k*_e_ = *εk*_g_ + (1 − *ε*)*k*_s_

where *k*_g_ and *k*_s_ are the thermal conductivities of the gas and solid catalyst phases, respectively. *C*_p,g_ is the specific heat capacity of the gaseous mixture and can be found using the average mass for the individual components:40
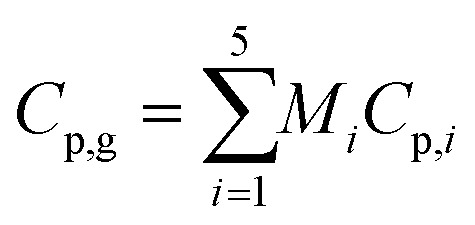


The boundary conditions obtained for the packed bed microreactor model are as follows:41
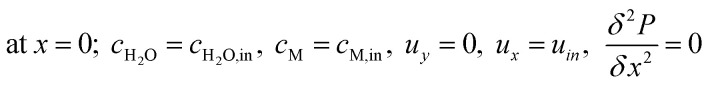
42
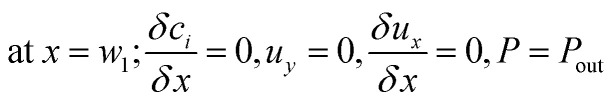
43
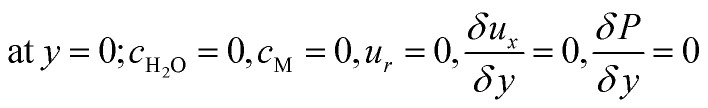

44 at *r* = 1; *c*_*i*,p_ = *c*_*i*,ps_

45
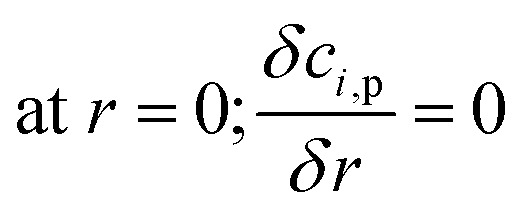


The boundary conditions obtained for the coated wall microreactor model are as follows:46
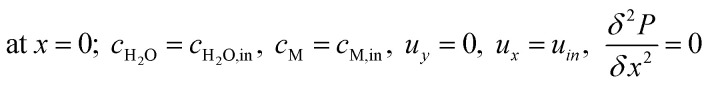
47
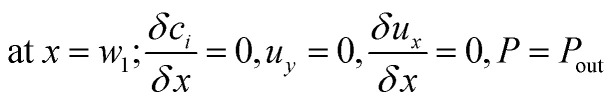
48
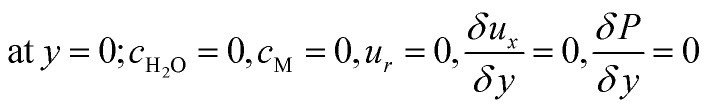

49 at *r* = 1; *c*_*i*,p_ = *c*_*i*,ps_

50
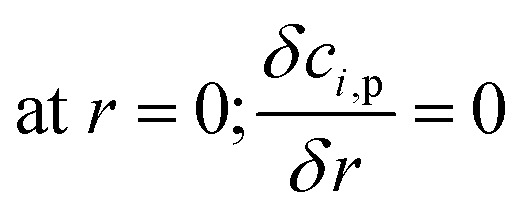

51 at *y* = *h*_1_; *c*_H_2_O,b_ = *K* × *c*_H_2_O_, *c*_M,b_ = *K* × *c*_M_

52 at *y* = *h*_2_, *c*_H_2_O,b_ = *K* × *c*_H_2_O_, *c*_M,b_ = *K* × *c*_M_

The mass balance equations coupled with the appropriate boundary conditions were solved using COMSOL Multiphysics® software version 5.3. A grid sensitivity analysis was performed to determine the effect of the mesh size on the accuracy of the resulting numerical solution. The grid numbers tested for the packed bed microreactor were 568000, 1136000 and 1704000, the numbers tested for the coated wall microreactor were 684154, 1368308, 2052462. The resulting mole fractions of CH_3_OH and H_2_ at the reactor outlets were observed to determine the effects of the mesh size (Table 1). It can be observed that for grid numbers of 568000 and 684154 from the packed bed and coated wall microreactors respectively generated the smallest difference between the tested numbers. As a result, the completed geometry for the packed bed microreactor comprised of a mesh consisting of 568000 domain elements and 32265 boundary elements, and 108254 degrees of freedom was used, and the results were found to be mesh independent with a computational time of 7.5 seconds. The geometry for the coated wall microreactor comprised of a mesh consisting of 684154 domain elements and 56257 boundary elements, and 120300 degrees of freedom was used, and the results were found to be mesh independent with a computational time of 8 seconds. The 3-D geometry for the packed bed consisted of 976703 domain elements with a computational time of 16 seconds, and the 3-D geometry for the coated wall reformer comprised of 989852 domain elements and a computational time of 18 seconds. Table 2 shows the parameters used for the CFD modelling study.

Grid sensitivity study for the packed bed and coated wall microreactors. *T*_w_ = 210 °C, S/M = 1.1, packed bed 75 μm pellet model, coated wall thickness 100 μm

**Table:** 

Packed bed microreactor			
Number of elements	568000	1136000	1704000
*y*_CH_3_OH_	0.1633	0.1633	0.1647
*y*_H_2__	0.4124	0.4123	0.4125

**Table:** 

Coated wall microreactor			
Number of elements	684154	1368308	2052462
*y*_CH_3_OH_	0.1426	0.1425	0.1426
*y*_H_2__	0.3312	0.3312	0.3324

**Table:** Parameters used for the CFD modelling studies

Symbol	Value	Units	Description
*c*_g_	*P*_g_/*RT*	mol m^−3^	Concentration of reacting gases
SMR	1.1	—	Steam-methanol molar ratio^20^
*l*_1_	1.5 × 10^−3^	m	Height of packed bed^20^
*w*_1_	0.9–1.1 × 10^−2^	m	Catalyst loading length of packed bed^20^
*m*_c_	1.5–1.6 × 10^−3^	kg	Mass of catalyst^20^
*h*_1_	100	μm	Coating thickness of catalyst^20^
*ν*	2−30 × 10^−3^	mL min^−1^	Inlet flow rate^20^
*v*_L_	0.1	m s^−1^	Inlet velocity
*T*	473–523	K	Reaction temperature^20^
*d*_pe_	7.5 × 10^−5^ to 1.5 × 10^−4^	m	Radius of catalyst pellet (packed bed)^20^
*V*_p_	4/3π*r*_pe_	m^3^	Volume of pellet
*ε*	0.4	—	Catalyst bed porosity
*ρ*_b_	1300	kg m^−3^	Catalyst density^23^
*k*_e_	0.3	W m^−1^ K^−1^	Thermal conductivity of catalyst^15^
*D*_M_	6.8 × 10^5^	m^2^ s^−1^	Bulk diffusion coefficient
*R*	8.314	J mol^−1^ K^−1^	Ideal gas constant
*A*_R_	1.15 × 10^6^	m^3^ s^−1^ kg^−1^	Amphletts constant^22^
*B*_R_	9.41 × 10^5^	m^3^ s^−1^ kg^−1^	Amphletts constant^22^
*A*_D_	7.09 × 10^7^	m^3^ s^−1^ kg^−1^	Amphletts constant^22^
*E*_R_	84 100	J mol^−1^	Reforming reaction activation energy^22^
*E*_D_	111 200	J mol^−1^	Decomposition reaction activation energy^22^

## 3. Results and discussion

### 3.1 Model validation

**Fig. 2:**
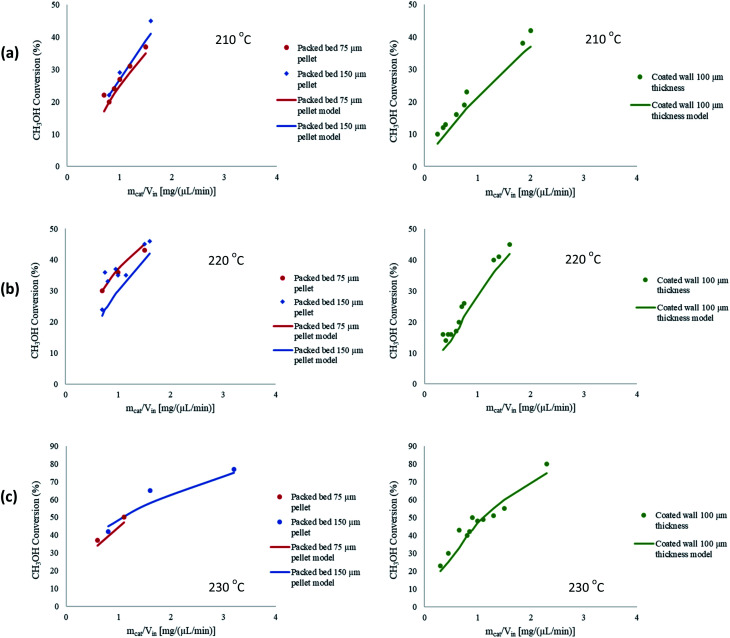
Comparison of coated wall and packed bed microreactors at different catalyst sizes and wall temperatures with experimental^20^ results: (a) *T*_w_ = 210 °C, S/M = 1.1; (b) *T*_w_ = 220 °C, S/M = 1.1; (c) *T*_w_ = 230 °C, S/M = 1.1.

The CFD microreactor models were compared with experimental data^20^ to assess the validity of the models. Fig. 2 shows the comparison between the packed bed and coated wall microreactor at three different reaction temperatures, and varying catalyst particle sizes. The parameter *m*_cat_/*V*_in_ is proportional to the residence time of the microreactors. The results show that the methanol conversion increases with increasing temperature. In addition, increasing the catalyst loading (*m*_cat_) also enhances the methanol conversion. It can be observed that there is a small difference in methanol conversion between the 75 μm and 150 μm catalyst particles, and so, it appears that there is a negligible effect of catalyst particle size on conversion. Similar results were obtained by Jiang *et al.*^34^ in which they performed methanol reforming with the CuO/ZnO/Al_2_O_3_ based catalyst (BASF S3-85) catalyst. The catalyst particle sizes varied from 150–590 μm and the results showed that there was no effect on the catalyst size on the conversion. It can be remarked that there is a good agreement in results between the experimental (literature) and the CFD models. A percentage discrepancy of less than 5% was observed between the experimental and modelling values.

Typically, the methanol conversion would be controlled by the temperature distribution. This means that for constant wall temperatures, the average temperature of the catalyst layer in the coated wall reactor should generate higher conversions when compared to the packed bed reactor. This was demonstrated by Bravo *et al.*^35^ who compared the performance of coated wall and packed bed reformers which were 4.1 mm in diameter. The results showed that at a reaction temperature of 230 °C, the coated wall reformer produced higher methanol conversions than the packed bed reformer using a CuO/ZnO/Al_2_O_3_ (BASF F13456) catalyst. For the present study, there is a very little difference in conversions between the packed bed and coated wall reformer. This could owe to the fact that the reformers used in this study were significantly smaller than the 4.1 mm used by Bravo *et al.*;^35^ hence, the temperature difference in the 1.5 mm diameter packed bed reactor is less the that for the reformer used by Bravo *et al.*^35^ As a result, temperature differences between the packed bed and coated wall microreactors used can be deemed negligible. It can be concluded that the performance of the packed bed and coated wall microreactors are similar under the current conditions.

**Fig. 3:**
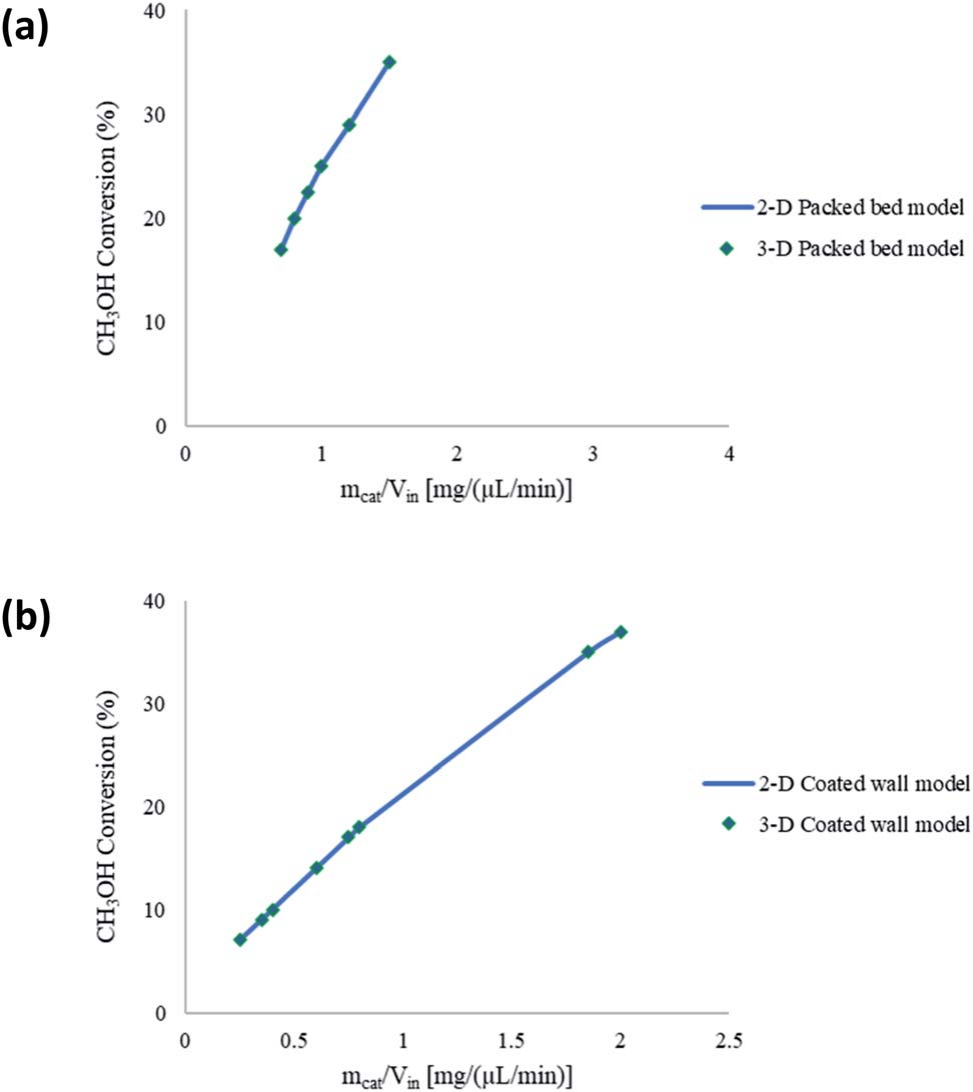
Comparison between 2-D and 3-D modelling configurations for (a) packed bed, and (b) coated wall microreactors. *T*_w_ = 210 °C, S/M = 1.1, packed bed 75 μm pellet model, coated wall thickness 100 μm.

A further study was performed to test the robustness and validity of the model by comparing the performance of 2-D and 3-D modelling configurations. With regards to 3-D modelling there is an additional spatial direction to solve the reactor parameters, and the reactor is of a cylindrical geometry. Fig. 3(a) shows the comparison between the two configurations for the packed bed microreactor, whilst Fig. 3(b) shows this comparison between the coated wall microreactor. The results depict a negligible difference in performance between the 2-D and 3-D models. The geometry of the microreactors eliminate gradients, such as temperature, thus, 2-D modelling was applied throughout the whole study. The 2-D modelling configuration also have a lower computational time to solve the defined problem. In addition, the width of the microreactors are larger than the height making them further suited to 2-D configurations. Similar findings were observed by Guo *et al.*^36^ whereby both 2-D and 3-D modelling generated similar results. Therefore, 2-D modelling was used for the whole investigation of electrochemical simulations. Furthermore, Cutress *et al.*^37^ analysed the commercial general engineering finite element software in electrochemical simulations and concluded that 2-D problems are within an order of magnitude of accuracy of finite difference simulations and analytical solutions, as long as the problem is well defined in the software and care is taken with regards to appropriate meshing and boundary conditions.

**Fig. 4:**
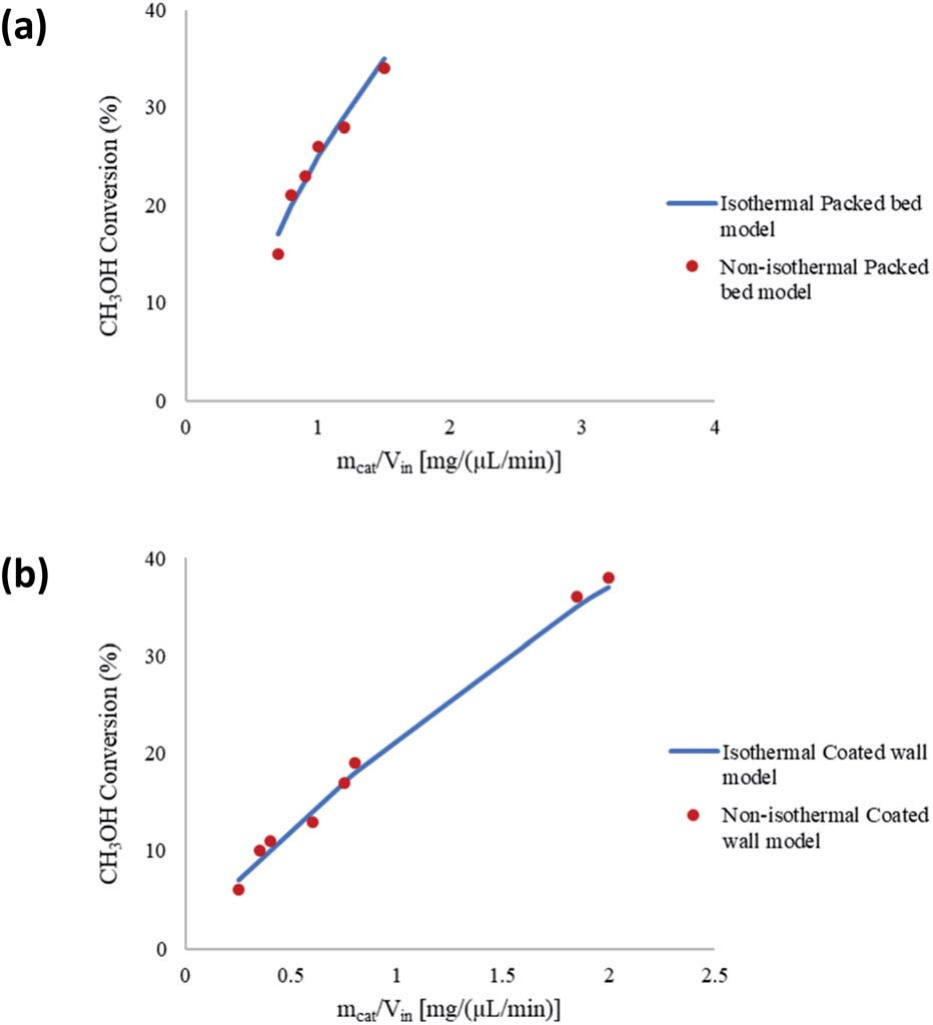
Comparison between isothermal and non-isothermal modelling conditions for (a) packed bed, and (b) coated wall microreactors. *T*_w_ = 210 °C, S/M = 1.1, packed bed 75 μm pellet model, coated wall thickness 100 μm.

The microreactors used in this study for the steam reforming of methanol operate isothermally. Nonetheless, non-isothermal configurations for both the packed bed and coated wall microreactor are investigated to determine any effects this may have on the conversion of CH_3_OH. Fig. 4 shows a comparison between isothermal and non-isothermal conditions for (a) packed bed, and (b) coated wall microreactors. The results show that there are negligible differences between the different modelling configurations, and so isothermal conditions were continued to be assumed throughout the whole study. The experimental data^20^ found similar results between the packed bed and coated wall reformers. This coincides with the findings reported in Fig. 2 demonstrating that the packed bed and coated wall microreactors perform similarly. The characteristically small dimensions of the microreactors enhance the heat transfer and can potentially diminish any temperature gradients which may exist in larger conventional reactors. Other reactions occurring in microreactors have also reported analogous findings regarding the isothermality of microreactors such as, aerobic oxidations.^38,39^ These reactions are highly exothermic, for example, the oxidation of benzyl alcohol to benzaldehyde has an overall heat of reaction of −187 kJ mol^−1.^^40^ However, these exothermic reactions were regarded as isothermal in microreactors due to the reactor's small dimensions and enhanced heat transfer.

### 3.2 Effect of flow

The packed bed microreactor was modelled using the laminar flow behaviour and assumptions. In order to achieve plug flow conditions of the reacting fluids through the catalyst bed, the diameter of the catalyst particle should be less than 0.1 times the inner diameter of the reactor.^41^ Such conditions can often be met in microreactor systems; however, may not be easily met in conventional systems due to large pressure drops across the reactor. Laminar flow behaviour was determined by obtaining the Re number in packed beds using the correlation:^42^53
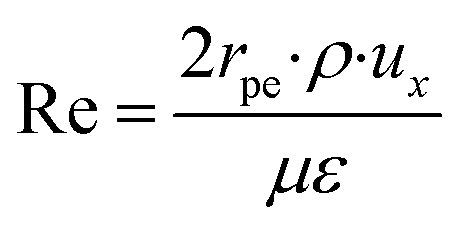


**Fig. 5:**
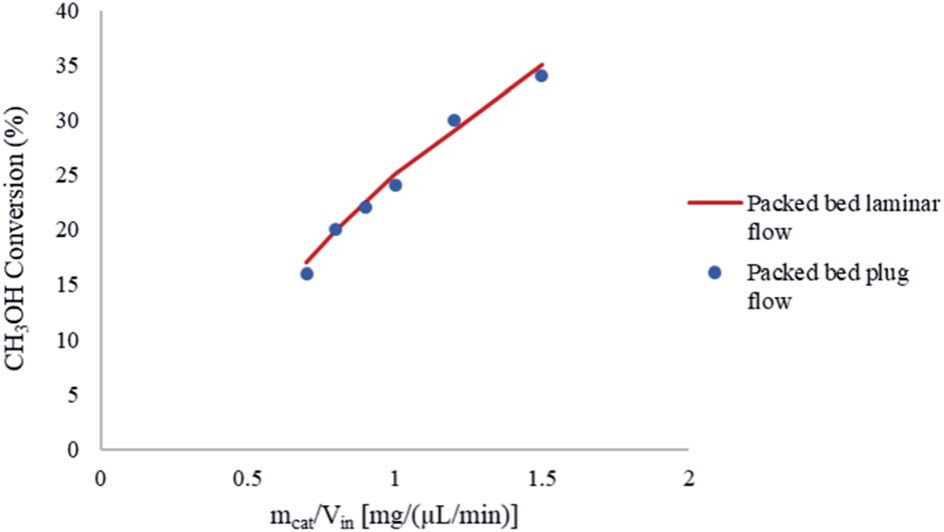
Effect of laminar and plug flow on the conversion of CH_3_OH in the packed bed microreactor. Methanol–water flow rate = 10 μL min^−1^, *T*_w_ = 210 °C, S/M = 1.1.

Fig. 5 shows the effects of the laminar and plug flow conditions on the conversion of CH_3_OH in the packed bed microreactor. It can be observed that there is a negligible difference in CH_3_OH conversion under the same conditions, and so the results are not affected by laminar or plug flow velocity profile. Therefore, in this modelling study, plug flow conditions can be assumed for the methanol steam reforming reaction in microreactors used for this study.

### 3.3 Component molar fractions

**Fig. 6:**
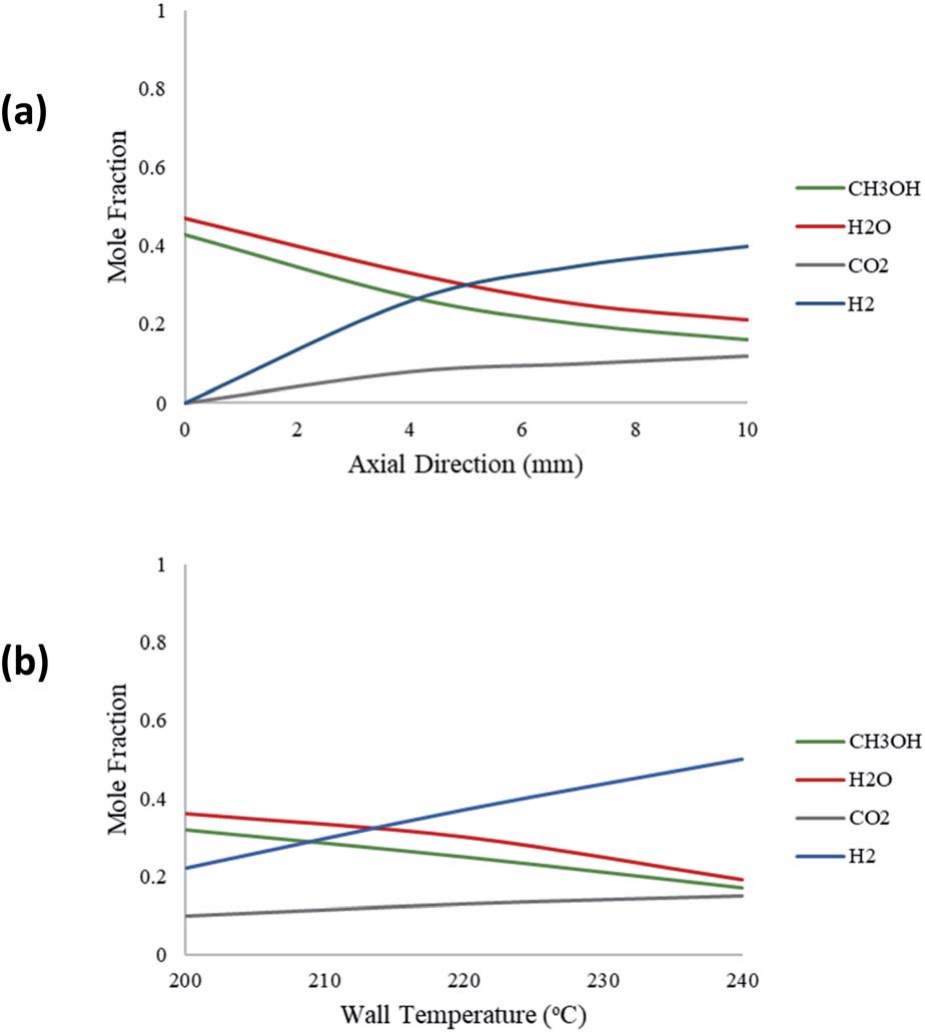
(a) Molar fraction variations of the components along the axial direction of the packed bed microreactor: methanol–water flow rate = 10 μL min^−1^, *T*_w_ = 220 °C, S/M = 1.1; (b) molar fraction variations of species at the packed bed microreactor outlet with respect to *T*_w_: methanol–water flow rate = 10 μL min^−1^, S/M = 1.1.

Fig. 6 depicts the molar fraction variations of the species reacted and produced for an inlet steam-methanol feed of 10 μL min^−1^. Fig. 6(a) shows this variation along the axial length of the packed bed microreactor for a constant temperature of 220 °C. The results show that the predominant products are H_2_ and CO_2_, with H_2_ having the greatest yield. There is also a negligible amount of CO produced which is incomparable with the other product yields. Furthermore, as the reaction progresses along the length of the microreactor, the product yields of H_2_ and CO_2_ also increase. Fig. 6(b) demonstrates the molar variations of the species at the outlet of the packed bed reactor with respect to varying wall temperatures. The results show that as the wall reforming temperature increases, the yield of the products also increases, as more heat is available to the reaction.

### 3.4 CO concentration

**Fig. 7:**
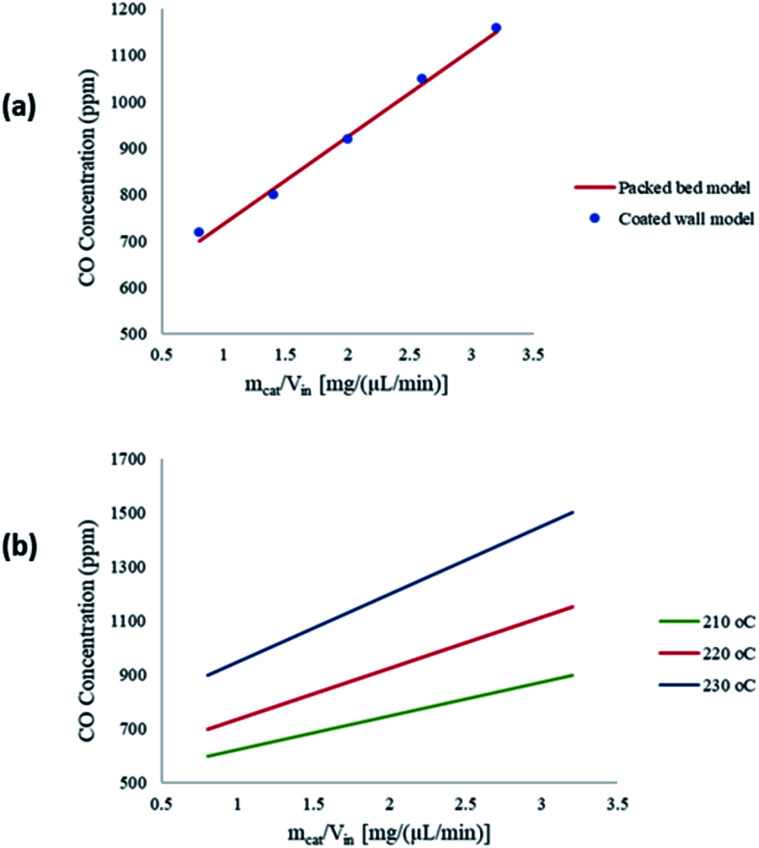
(a) Carbon monoxide produced in the packed bed and coated wall microreactors; (b) carbon monoxide produced during the reaction with respect to *m*_cat_/*V*_in_ at different wall temperatures in the packed bed microreactor. Methanol–water flow rate = 10 μL min^−1^, S/M = 1.1.

Fig. 7(a) shows a comparison between the amount of CO produced from the packed bed and coated wall microreactors at a reaction temperature of 220 °C. The results show that similar concentrations of CO are obtained at constant residence times. The findings suggest that the average temperatures of the packed bed are comparable to the coated wall microreactor, therefore temperature effects within the reformers are negligible. Fig. 7(b) depicts the CO concentration produced at three different wall temperatures in the packed bed microreactor. It can be observed that CO concentration increases with respect to the residence time in the microreactor. As the temperature increases, the level of CO concentration produced also increases. According to Amphlett *et al.*^22^ a small proportion of the methanol decomposes to produce CO and H_2_, and CO is also produced from the reverse water–gas-shift reaction. However, studies have shown that CO is mainly produced from the reverse water–gas-shift reaction.^20^

### 3.5 Effect of SMR

**Fig. 8:**
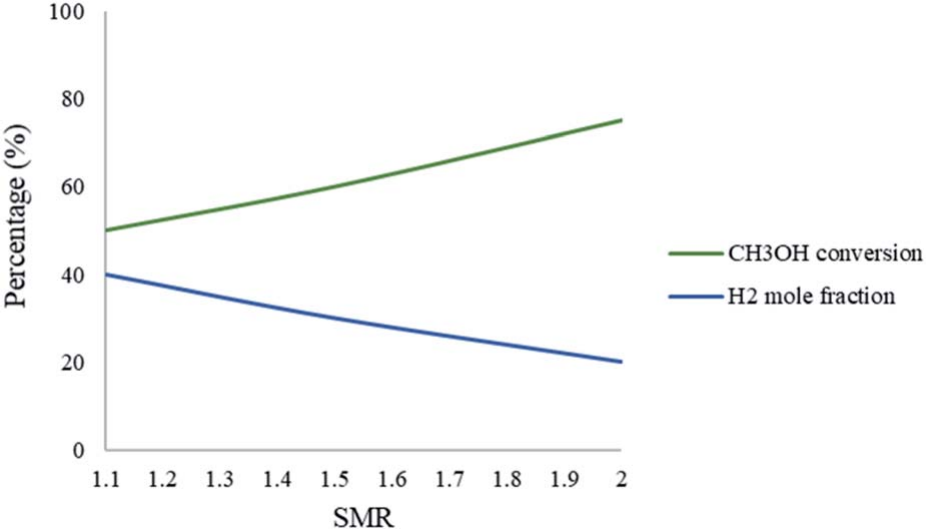
Effect of SMR on the conversion of CH_3_OH and the mole fraction of H_2_ in the packed bed microreactor. Methanol–water flow rate = 10 μL min^−1^, *T*_w_ = 220.

Fig. 8 represents the effect of the SMR on the conversion and production of CH_3_OH and H_2_, respectively. The results show that as the ratio increases in the packed bed reformer, the CH_3_OH conversion also increases; however, the mole fraction of H_2_ decreases. The SMR can have a significant effect on CH_3_OH conversion and increasing the ratio will enhance the conversion. The increase in ratio means that there is a higher amount of steam in the fluid stream, which in turn leads to the dilution of the H_2_ in the product stream. A SMR value of 1.1 is a compromise between these observations.

### 3.6 Effect of catalyst coating thickness

**Fig. 9:**
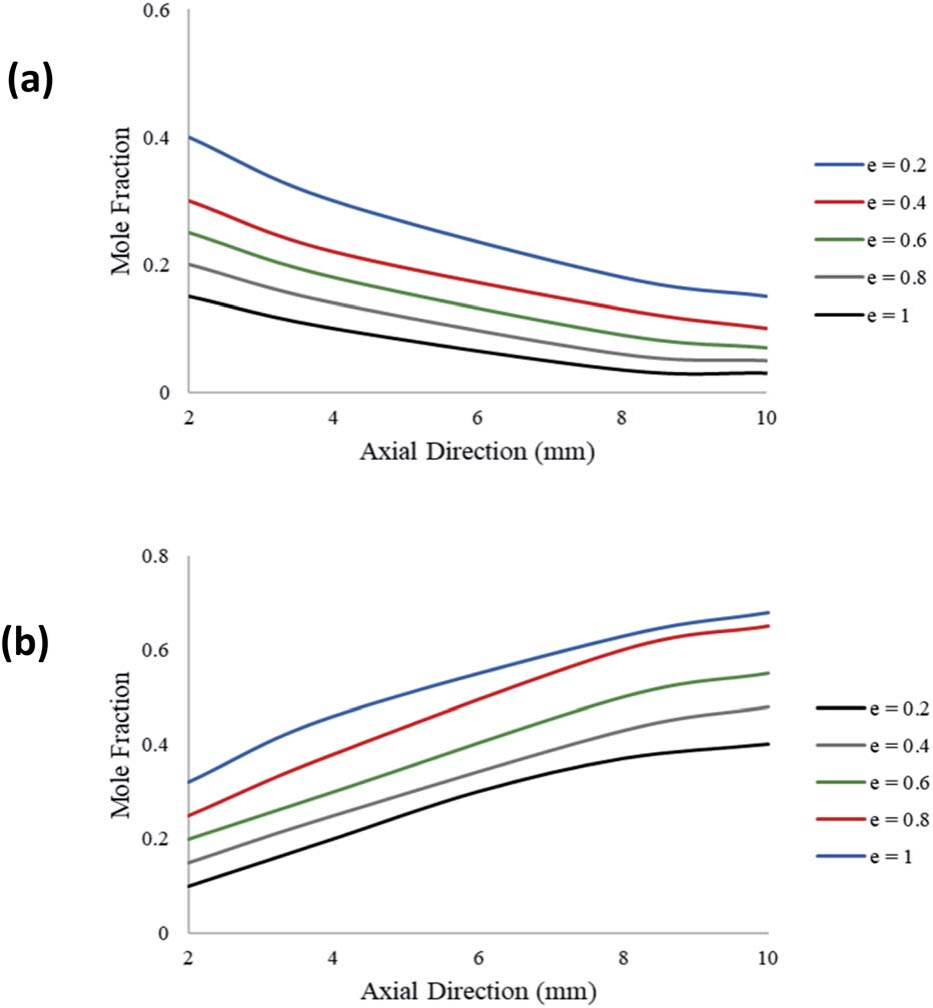
Molar variations of (a) CH_3_OH; and (b) H_2_; for varying catalyst thickness along the axial direction of the coated wall microreactor. Methanol–water flow rate = 10 μL min^−1^, *T*_w_ = 220 °C, S/M = 1.1.

Fig. 9 demonstrates the effect of catalyst layer thickness on the molar fraction of CH_3_OH and H_2_ along the axial direction of the coated wall microreactor. The variable *e* represents the ratio of the catalyst layer thickness against the height of the reformer. A packed bed configuration would represent a ratio of *e* = 1. It can be seen from Fig. 9(a) that as the catalyst coating thickness increases, the CH_3_OH conversion also increases. Furthermore, the decline in CH_3_OH concentration is greatest towards the inlet of the reactor, as the reaction progresses along the axial direction the change in concentration becomes slight. This indicates that the rate of the steam methanol reforming reactions is greatest towards the region of the entrance due to the higher concentrations of the reacting fluids. Fig. 9(b) shows the change in H_2_ production at varying catalyst coating thicknesses. Again, a thicker catalyst coating results in a higher product yield of H_2_.

### 3.7 Study of mass transfer resistances

**Fig. 10:**
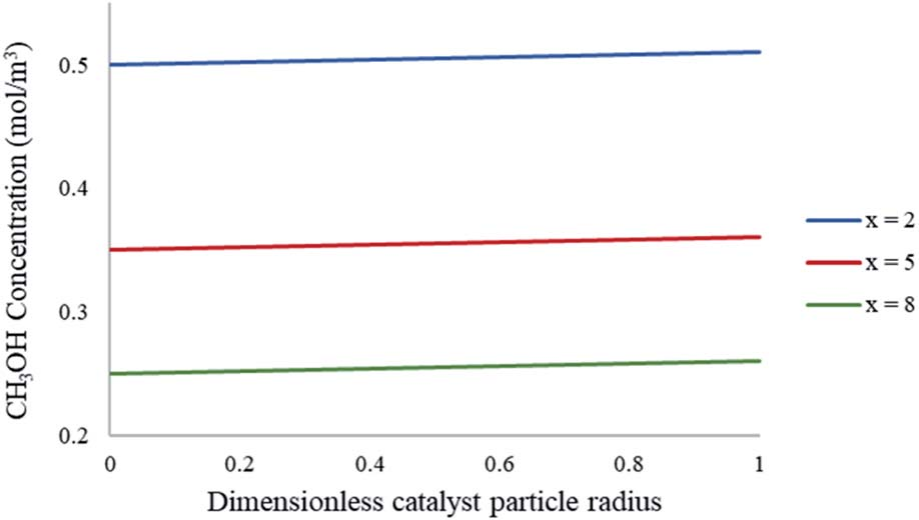
Concentration profiles of CH_3_OH within the catalyst particle at varying axial lengths of the packed bed microreactor. Methanol–water flow rate = 10 μL min^−1^, *T*_w_ = 220 °C, S/M = 1.1, packed bed 75 μm pellet model.

The CFD microreactor models consider the solid catalyst reacting heterogeneously with the reacting fluids. As a result, the models can determine the internal and external mass transfer limitations occurring within the microreactors. Factors which cause the reaction to be diffusion limited or surface-reaction-limited can be ascertained, enabling an understanding of how the methanol reforming process can be enhanced. Fig. 10 demonstrates the concentration profiles of CH_3_OH inside the catalyst pellet. This study was performed using the packed bed microreactor model at *y* = 0.5 mm, and different lengths of *x* = 2; 5 and 8 mm. The size of the catalyst particles inside the packed bed microreactor ranged from 75–150 μm. A steep concentration gradient would be the result of internal mass transfer limitations. From Fig. 10 it can be observed that the disparity from the surface of the catalyst pellet (*r* = *R*) to inside the pellet (*r* = 0) is lower than 5%, leading to the conclusion that there are no pore diffusion limitations present. Furthermore, additional studies were conducted to assess the pore diffusion limitations, whereby the catalyst particle sizes were halved and quartered whilst maintaining all other reactor properties constant. The results concluded that there was no substantial discrepancy (<1%) in the conversion of methanol. To further validate the CFD findings, the Thiele modulus (*ϕ*) was calculated for the particle sizes of 75–150 μm. According to a first-order reaction with solid spherical particles, the Thiele modulus can be given by:^32^54
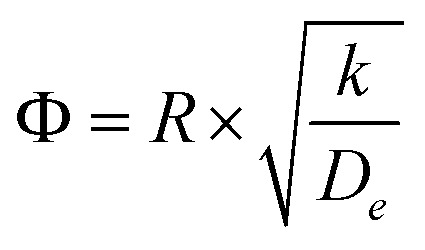
where *R* is the catalyst particle radius, *k* is the reaction rate constant and *D*_*e*_ is the catalyst particle diffusivity. For this reaction, the value of the Thiele modulus was found to be significantly less than 1 which corresponds to an effectiveness factor of unity. Therefore, it can be established that the reaction is surface-reaction-limited and that there are negligible pore diffusion limitations for this study. Larger values of the Thiele modulus demonstrate that the surface reaction is rapid, and that majority of the reactants would be consumed at the surface of the spherical pellet, leaving very little to penetrate the catalyst particle interior. The study has found that for very large values of the rate constant, the reaction appears to become diffusion limited. The lack of mass transfer resistances in microreactors elaborates their advantages.

**Fig. 11:**
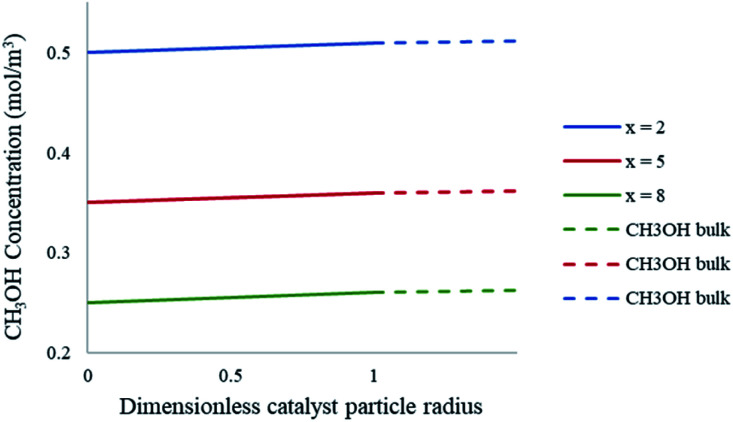
Concentration profiles of CH_3_OH at the catalyst particle surface and the bulk fluid at varying axial lengths of the packed bed microreactor. Methanol–water flow rate = 10 μL min^−1^, *T*_w_ = 220 °C, S/M = 1.1, packed bed 75 μm pellet model.

In order to determine the external mass transfer resistances, the concentration surrounding the catalyst pellet must be compared to that of the pellet surface. The methanol steam reforming reaction involves the mass transfer and diffusion of the reacting gases into the contiguous region of the catalyst pellet. Fig. 11 represents the bulk concentration of methanol compared to the surface of the catalyst particle in the packed bed microreactor. The results show that there is a less than 1% difference between the bulk concentration of reactant in the boundary layer when compared to the concentration on the pellet surface. As a result, there is negligible resistance to the diffusion crossing the boundary layer to the solid particle surface, hence no external mass transfer resistances present in the study.

One of the notable advantages of using microreactors for methanol steam reforming is the improved mass transfer they offer. A study of comparison was conducted between the current work and the experimental work by Purnama *et al.*^41^ in which a larger reactor was used. The steam reforming of methanol was investigated over a commercial CuO/ZnO/Al_2_O_3_ catalyst in a packed-bed reactor under atmospheric pressure and a reaction temperature of 230–300 °C. A tubular stainless-steel reactor with an internal diameter of 10 mm was packed with the solid catalyst which had a particle size of 0.71 and 1 mm. To assess the pore diffusion limitations, the Thiele modulus was calculated using eqn (54). The results showed that for this reaction the Thiele modulus was significantly greater than 1 which indicates the presence of internal mass transfer resistances.

**Fig. 12:**
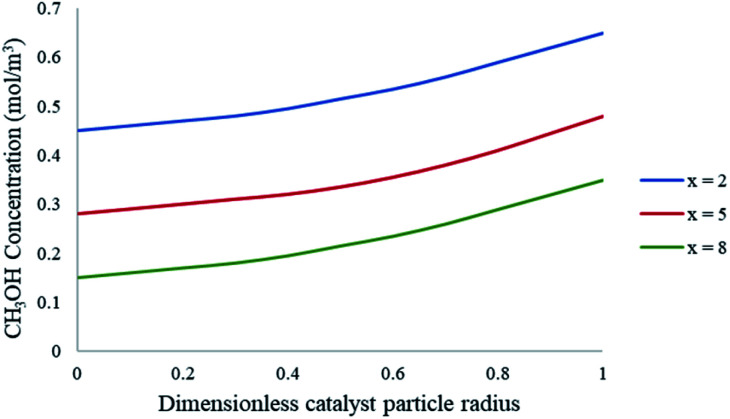
Concentration profiles of CH_3_OH within the catalyst particle at varying axial lengths of the packed bed microreactor. Methanol–water flow rate = 10 μL min^−1^, *T*_w_ = 220 °C, S/M = 1.1. Packed bed 0.75 mm pellet model.

**Fig. 13:**
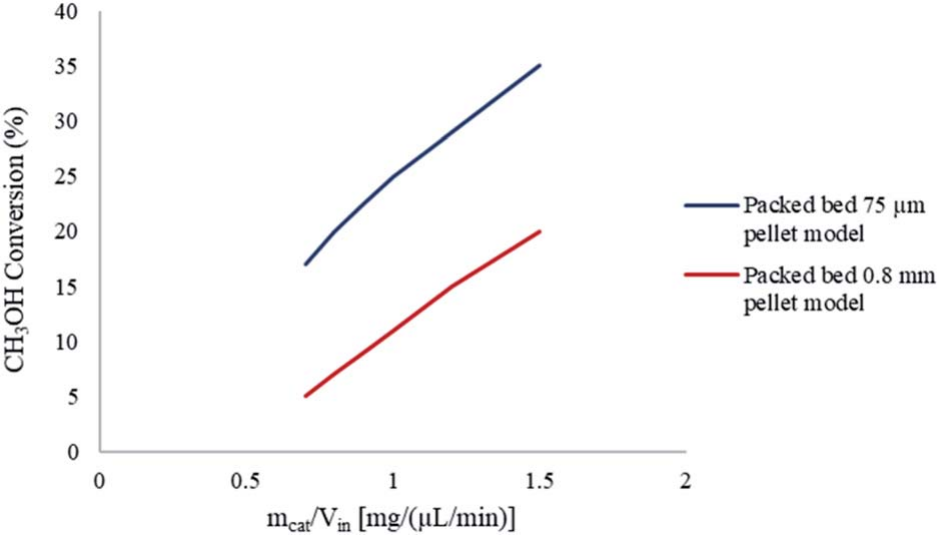
Effect of catalyst particle size on CH_3_OH conversion. Methanol–water flow rate = 10 μL min^−1^, *T*_w_ = 220 °C, S/M = 1.1.

To further understand the effects of these limitations on the methanol steam reforming reaction, the catalyst size used in the current study was increased to 0.75–1 mm to be comparable with that used by Purnama *et al.*^41^ Under these conditions, the concentration profile inside the catalyst pellet is shown in Fig. 12. The results were obtained at *y* = 0.5 mm, and different reactor lengths of *x* = 2; 5 and 8 mm. It can be concluded that larger catalyst particle sizes lead to an increase in pore diffusion limitations for the steam reforming of methanol. Fig. 13 demonstrates the effect of particle size on the conversion of CH_3_OH. The catalyst particle sizes used for this study were 75–150 μm and 0.75–1 mm. It can be concluded that under the current reaction conditions, larger catalyst sizes lead to internal mass transfer resistances, which in turn lead to a lower CH_3_OH conversion. In order to increase the reaction rate for reactions which are internally diffusion limited, the pellet radius can be decreased, the reaction temperature could be increased as well as the concentration, or the internal surface area should be increased.^32^

Pore diffusion limitations can be absent in a packed bed reactor if small sized catalyst pellets are used. Furthermore, higher CH_3_OH conversions can be achieved by using smaller pellets. However, the use of extremely small pellets can cause excessive pressure drops across conventional reactors. Microreactors often eliminate the issue of large pressure drops due to their small dimensions, as well as enhancing the mass transfer. The pressure drop in the packed bed microreactor was found to be less than 1 Pa, which can be considered insignificant.

## 4. Conclusions

The modelling results obtained in this study for the steam reforming of methanol over a CuO/ZnO/Al_2_O_3_ based catalyst (BASF F3-01) have shown a good validation with experimental results acquired from the literature. It was found that the methanol conversion increases with increasing temperature and residence time. The performance of the packed bed and coated wall reformer at a constant wall temperature was analogous, indicating that the average temperature of the catalyst bed in the packed bed microreactor and the average temperature of the catalyst layer in the coated wall microreactor are similar. The results from the packed bed reformer showed that difference in conversion between the 75 μm and 150 μm catalyst particles was insignificant. This seems to indicate that there are no limitations in the internal pore diffusion for the two catalyst particles. Moreover, the performance of the coated wall microreactor was analysed by investigating the size of the catalyst thickness. The results showed at higher catalyst thicknesses, the methanol conversion and hydrogen production were enhanced. The heterogeneous models were able to analyse the reaction-coupled transport phenomena occurring within the microreactor. A study of internal and external mass transfer limitations was performed by generating concentration profiles between the bulk fluid and within the catalyst particle. From the results, it was concluded that the microreactors used in this study are devoid of any internal and external mass transfer resistances. Furthermore, the results from the CFD were compared to a study which used a larger reactor. It was found that using larger catalyst particles led to internal mass transfer resistances. It was also concluded that the presence of these pore diffusion limitations caused lower methanol conversions, as opposed to smaller catalyst particle sizes used in microreactor systems which have no pore diffusion limitations and negligible pressure drops. The CFD models created in this study have the ability to predict the steam reforming of methanol for hydrogen production in microreactors. Microreactors are known for their enhanced mass and heat transfer, and the ability to be used in offshore remote locations amongst various other benefits, so future research could be directed towards investigating the scalability of these devices to produce hydrogen.

## Conflicts of interest

There are no conflicts to declare.

## Supplementary Material
